# Evaluating the Effect of Artificial Liver Support on Acute-on-Chronic Liver Failure Using the Quantitative Difference Algorithm: Retrospective Study

**DOI:** 10.2196/45395

**Published:** 2023-10-24

**Authors:** Tinghuai Huang, Jianwei Huang, Timon Cheng-Yi Liu, Meng Li, Rui She, Liyu Liu, Hongguang Qu, Fei Liang, Yuanjing Cao, Yuanzheng Chen, Lu Tang

**Affiliations:** 1 School of Physical Education and Sports Science South China Normal University Guangzhou China; 2 Department of Gastroenterology The Fifth Affiliated Hospital of Guangzhou Medical University Guangzhou China; 3 Civil Aviation Flight University of China Chengdu China

**Keywords:** double plasma molecular absorption system, DPMAS, acute-on-chronic liver failure, quantitative difference

## Abstract

**Background:**

Liver failure, including acute-on-chronic liver failure (ACLF), occurs mainly in young adults and is associated with high mortality and resource costs. The prognosis evaluation is a crucial part of the ACLF treatment process and should run through the entire diagnosis process. As a recently proposed novel algorithm, the quantitative difference (QD) algorithm holds promise for enhancing the prognosis evaluation of ACLF.

**Objective:**

This study aims to examine whether the QD algorithm exhibits comparable or superior performance compared to the Model for End-Stage Liver Disease (MELD) in the context of prognosis evaluation.

**Methods:**

A total of 27 patients with ACLF were categorized into 2 groups based on their treatment preferences: the conventional treatment (n=12) and the double plasma molecular absorption system (DPMAS) with conventional treatment (n=15) groups. The prognosis evaluation was performed by the MELD and QD scoring systems.

**Results:**

A significant reduction was observed in alanine aminotransferase (*P*=.02), aspartate aminotransferase (*P*<.001), and conjugated bilirubin (*P*=.002), both in *P* values and QD value (Lτ>1.69). A significant decrease in hemoglobin (*P*=.01), red blood cell count (*P*=.01), and total bilirubin (*P*=.02) was observed in the DPMAS group, but this decrease was not observed in QD (Lτ≤1.69). Furthermore, there was a significant association between MELD and QD values (*P*<.001). Significant differences were observed between groups based on patients’ treatment outcomes. Additionally, the QD algorithm can also demonstrate improvements in patient fatigue. DPMAS can reduce alanine aminotransferase, aspartate aminotransferase, and unconjugated bilirubin.

**Conclusions:**

As a dynamic algorithm, the QD scoring system can evaluate the therapeutic effects in patients with ACLF, similar to MELD. Nevertheless, the QD scoring system surpasses the MELD by incorporating a broader range of indicators and considering patient variability.

## Introduction

Liver failure, including acute-on-chronic liver failure (ACLF), occurs mainly in young adults and is associated with high mortality and resource costs [1,2]. Management of patients with liver failure aims to maintain or restore vital organ functions, prevent the development of multiorgan failure, and bridge them to recovery or transplantation until an appropriate donor organ becomes available. As an extracorporeal procedure, the double plasma molecular absorption system (DPMAS) combines broad-spectrum plasma adsorption with specific bilirubin adsorption, making it highly desirable to provide time for spontaneous liver regeneration or emergency liver transplantation to be undertaken. Two absorbers separated and cleaned toxic plasma during the procedure before returning it to the patients [3-5].

Meanwhile, the prognosis evaluation of liver failure should run through the entire diagnosis and treatment process, especially in the early prognosis evaluation. This involves using various methods, including the Child-Pugh classification [6], the indocyanine green excretion rate [7,8], the preoperative liver volume assessment, and the Model for End-Stage Liver Disease (MELD) [9-14]. However, each method has its limitations [15-19]. Although most of the prognostic models in hepatology, including MELD and Child-Pugh classification, were developed as static models, the full predictive potential of the dynamic trajectory of these models has received little attention so far [20]. In addition, the therapeutic effects in patients with liver failure can only be evaluated according to the level of toxins, transaminase activity, and coagulation function, and the results could be influenced by many factors, including age. Therefore, it is crucial to establish a novel approach to rapidly, accurately, and objectively evaluate therapeutic efficacy of ACLF.

As a recently proposed novel algorithm, the quantitative difference (QD) algorithm is based on the ratio response of the Weber law in psychology and the Weber-Fechner law in molecular biology [21-23]. By drawing from these principles, the QD algorithm can detect the presence of differences among multiple data sets and quantify the magnitude of the disparity between 2 specific data sets. Therefore, the QD algorithm may hold immense value for medical applications, particularly in evaluating the treatment’s effectiveness in patients with ACLF, given the variability of factors, such as age, gender, and liver function.

In this study, the quantitative difference (QD) algorithm is introduced to evaluate and analyze the effect of DPMAS and conventional treatment in patients with ACLF. The objective is to examine whether the QD algorithm exhibits comparable or superior functionality compared to the MELD in the context of prognosis evaluation.

## Methods

### Patients and Setting

**Figure 1:**
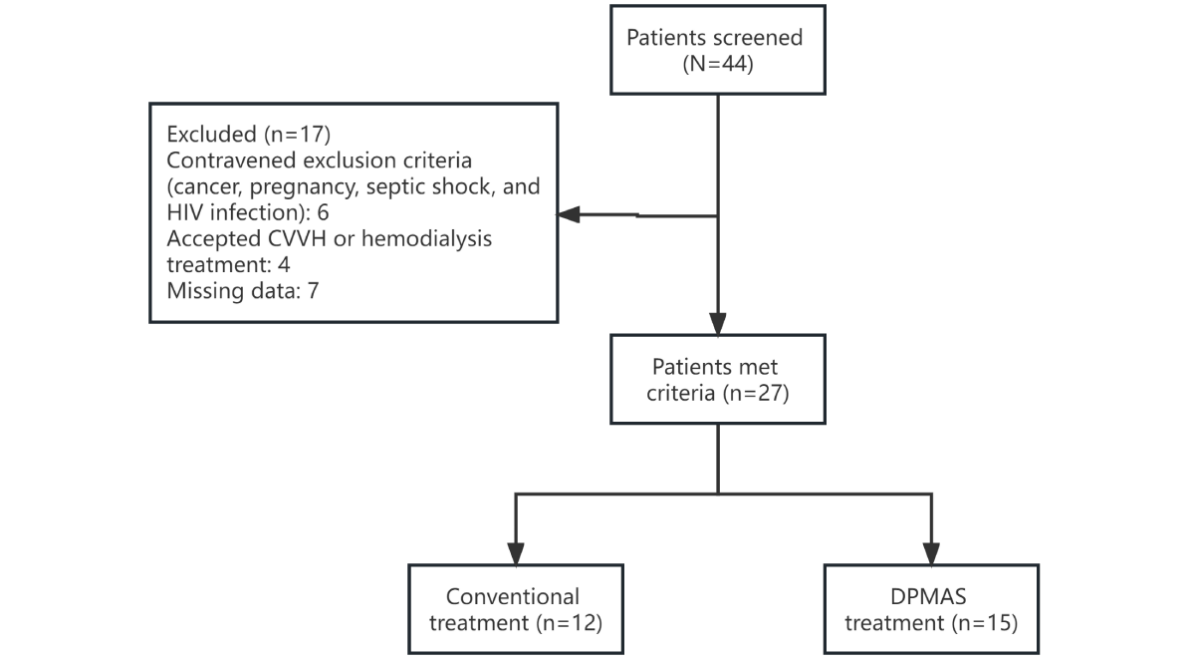
Study flow diagram. CVVH: continuous veno-venous hemofiltration; DPMAS: double plasma molecular adsorption system.

A single-center retrospective study was conducted to screen hospitalized patients in the Fifth Affiliated Hospital of Guangzhou Medical University between January 2018 and December 2020. The inclusion criteria for patients with ACLF were as follows: (1) meeting the diagnostic criteria for ACLF defined by the Asian Pacific Association for the Study of the Liver [24] and (2) aged 18-80 years. Among the 44 patients included in this study, 17 were excluded, most commonly due to contravening exclusion criteria (n=10) or lack of data (n=7). This left 27 patients with ACLF, who were categorized into the following 2 groups according to the treatment they chose to receive: (1) the DPMAS group, where patients received dialysis with DPMAS as well as conventional treatment (n=15) and (2) the conventional treatment group, where patients received conventional treatment alone (n=12). The formulation, implementation, and diagnosis of all patients were carried out under the regulations of the Fifth Affiliated Hospital of Guangzhou Medical University (Figure 1).

### The DPMAS Treatment

**Figure 2:**
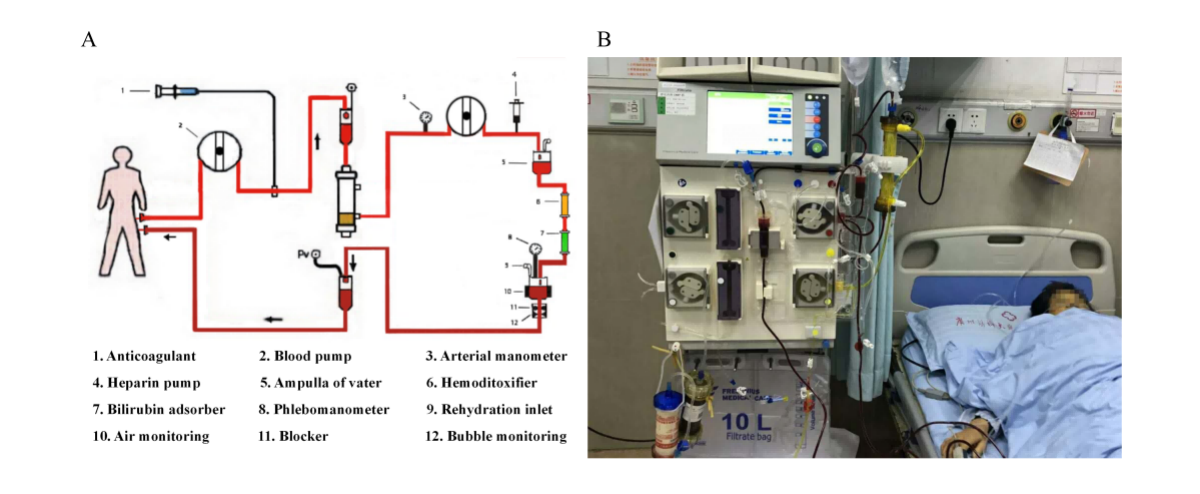
The DPMAS treatment.

**Figure 3:**
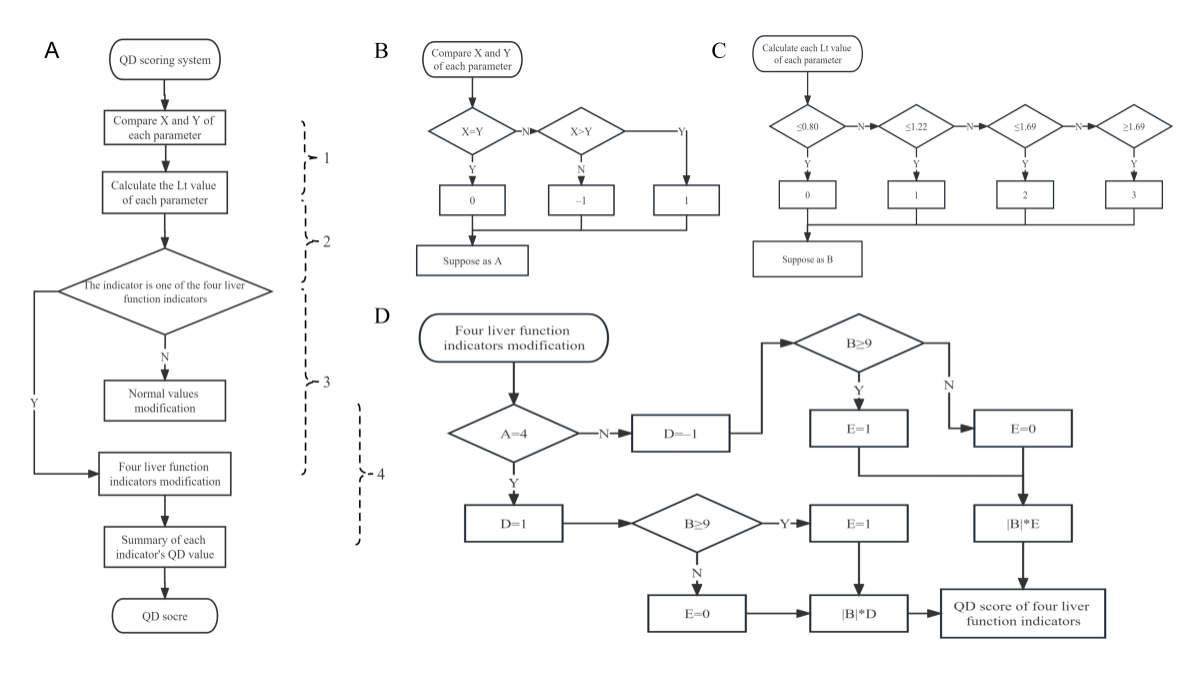
QD evaluation system procedure.

Patients were studied during a single 2-hour DPMAS treatment. The extracorporeal blood and plasma separation flow were maintained at 150 mL/min and 50 mL/min, respectively. A 5.2 version extracorporeal machine equipped with P2 plasma flux dry, MG350 hemoperfusion, and DX350 bilirubin adsorption column (all from Boxin biotechnology Co) were used to remove toxic molecules (Figures 2 and 3). The number of treatments was variable but limited to 16. Treatment was terminated if an organ became available for transplantation, if there was a significant clinical improvement, if the patient experienced marked deterioration, if there was an important adverse event, or if the patient died.

### Conventional Treatment (Both Groups)

Conventional treatment was standardized for each patient with ACLF. Cerebral edema was treated with head-of-bed elevation, prevented hepatic encephalopathy, controlled hypoproteinemia, and hypothermia. Hemorrhage and disseminated intravascular coagulation were treated with coagulation factor replacement (vitamin K1, fibrinogen, or fresh frozen plasma). Patients in the conventional treatment group received intensive critical care according to the current standard best practices at each study site. All patients were assessed for clinical status assessments every 12 hours.

### A Novel Scoring System

As a gold standard of statistical validity, *P* values are considered unreliable by many scientists, as they can only indicate the presence of differences between 2 data groups but do not provide information about how big these differences are [25-27]. Therefore, we introduce the QD algorithm to analyze treatment efficacy in patients with ACLF. The QD algorithm is based on the ratio response of the Weber law in psychology and the Weber-Fechner law in molecular biology [21,22]. In light of the Weber law, the concept of Weber threshold highlights a minimum value in the ratio between an objective parameter and its corresponding base value. Fechner extended the Weber law to create the Weber-Fechner law, which asserts that the relationship between objective parameters and the corresponding subjective parameters is logarithmic in nature. The change of subjective parameters corresponds to the logarithm of the ratio of objective parameters [28], as follows:

S = K_0_ log_a_ x (1)

The golden section constant Lτ is the basic natural unit that measures the ratio response. Liu [29] introduced the logarithm to the base of ^τ^ Lτ:



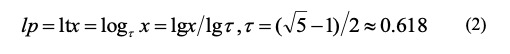



The concept of the QD can be approached from the perspective of self-similarity. Self-similarity was studied in the fractal literature, where a pattern is considered self-similar if it does not vary across different spatial or temporal scales [29,30]. It was found that there are QD thresholds (α and β) at various levels, including the cellular, molecular, or central nervous system levels (thresholds 0.80 and 1.22), at the organs or tissue level (thresholds 0.47 and 0.80), and the level of the body (thresholds 0.27 and 0.47). At the level of molecules, there are 3 levels of β: health level (β_1_=0.80), subhealth level (β_2_=1.22), and disease level (β_3_=1.69).

### The MELD Scoring System

Numerous studies have demonstrated the prognostic ability of the MELD scoring system [31]. Zhou et al [32] indicated that MELD could categorize patients according to their risk scores, distinguish the outcome of patients, and forecast survival in patients with ACLF. It incorporates 3 widely available laboratory variables, including the international normalized ratio [23], serum creatinine, and serum bilirubin. The original mathematical formula for MELD is as follows:


MELD = 9.57 × Log_e_(creatinine) + 3.78 × Log_e_(total bilirubin) + 11.2 × Log_e_(international normalized ratio) + 6.43 (3)

The higher the MELD score, the higher the short-term mortality risk. In this study, we also used MELD to evaluate the therapeutic effects of 2 different kinds of treatment to verify the feasibility and accuracy of the novel statistical model.

### Ethical Considerations

The study was reviewed and approved by the Ethics Committee of the Fifth Affiliated Hospital of Guangzhou Medical University (GYWY-L2021-31). All research data are processed anonymously.

## Results

### Overview

In the DPMAS group, 4 patients received a short session, 1 died during the treatment, and the remaining 10 were recovered and discharged. In the conventional treatment group, 2 patients were healed and discharged, and 3 died during the treatment, leaving 7 patients who gave up attending the treatment sessions.

Table 1 summarizes the two groups’ ages as well as the MELD and biochemical variables before treatment. There was no significant difference in both groups before and after treatment, except for activated partial thromboplastin time (*P*=.02), fibrinogen (*P*=.046), conjugated bilirubin (*P*=.046), and uric acid (*P=*.04).

**Table 1:** Postoperative data of the study participants.

Variable	Conventional treatment group (n=12), mean (SD)	DPMAS^a^ group (n=15), mean (SD)	*P* value
Age (years)	50.42 (14.88)	48.53 (10.17)	.70
MELD^b^	19.09 (7.88)	26.13 (9.43)	.06
Hemoglobin (g/L)	115.42 (31.44)	112.47 (22.42)	.78
White blood cell count (×10^9^/L)	9.03 (6.06)	9.29 (4.99)	.91
Red blood cell count (×10^12^/L)	4.2 (1.49)	3.98 (1.75)	.74
Platelet (×10^9^/L)	111.42 (80.54)	102.49 (66.87)	.76
Thrombocytocrit	0.18 (0.11)	0.12 (0.07)	.17
Activated partial thromboplastin time (s)	42.87 (11.57)	53.44 (10.79)	.02
Prothrombin time (s)	18.83 (4.74)	23.71 (7.09)	.05
Thrombin time (s)	21.56 (3.53)	33.51 (33.21)	.23
Fibrinogen (g/L)	2.16 (1.45）	1.32 (0.5)	.05
International normalized ratio	1.64 (0.43)	3.21 (4.44)	.24
Uric acid (μmol/L)	354.37 (143.16)	228.38 (140.69)	.04
Creatinine (μmol/L)	131.9 (109.8)	109.4 (69.88)	.53
Urea (mmol/L)	8.83 (10.98)	8.1 (8.8)	.86
Glucose (mmol/L)	7.11 (1.6)	8.47 (3.63)	.28
Alanine aminotransferase (U/L)	601.21 (1109.7)	689.4 (966.91)	.83
Aspartate aminotransferase (U/L)	419.21 (570.33)	595.31 (535.02)	.42
Total bilirubin (μmol/L)	205.73 (166.14)	378.29 (196.53)	.02
Conjugated bilirubin (μmol/L)	125.43 (134.49)	233.3 (130.71)	.05
Unconjugated bilirubin (μmol/L)	67.41 (55.92)	120.59 (94.63)	.11
Cholinesterase (U/L)	3536.32 (2107.08)	3695.99 (1515.02)	.83
γ-glutamyl (U/L)	341.4 (324.48)	192.02 (224.2)	.33

^a^DPMAS: double plasma molecular adsorption system.

^b^MELD: Model for End-Stage Liver Disease.

### Changes in Therapeutic Indicators

Biochemical variables are listed in Table 2. In the DPMAS group, there was a significant reduction in alanine aminotransferase (*P*=.02), aspartate aminotransferase (*P*<.001), and conjugated bilirubin (*P*=.002) both in *P* values and QD values (Lτ>1.69). A significant decrease in hemoglobin (*P*=.01), red blood cell count (*P*=.01), and total bilirubin (*P*=.02) was observed in the DPMAS group, but no significant decrease was observed in QD values (Lτ≤1.69). Nevertheless, all indicator values remained unchanged, both in *P* and QD values (Lτ≤1.69). In other words, the *P* value supports the conclusions drawn by the QD algorithm, indicating that the algorithm and the thresholds we have chosen are suitable for evaluating the therapeutic efficacy of ACLF.

**Table 2:** Preoperative and postoperative data of the double plasma molecular adsorption system (DPMAS) group and conventional treatment group.

Variable	DPMAS group, mean (SD)					Conventional treatment group			
	Preoperative data, mean (SD)	Postoperative data, mean (SD)	*P* value	QD^a^ value	Preoperative data, mean (SD)		Postoperative data, mean (SD)	*P* value	QD value
Hemoglobin (g/L)	112.47 (22.42)	89.53 (23.11)	.01	0.502	115.42 (31.44)		102.91 (28.49)	.33	0.244
White blood cell count (×10^9^/L)	9.29 (4.99)	11.22 (5.82)	.35	0.414	9.03 (6.06)		8.33 (3.72)	.74	0.085
Red blood cell count (×10^12^/L)	3.98 (1.75)	2.66 (0.77)	.01	0.791	4.19 (1.49)		3.38 (1.12)	.16	0.427
Platelet (×10^9^/L)	102.49 (66.87)	106.27 (67.23)	.88	0.249	111.42 (80.54)		124.33 (92.42)	.72	0.111
Thrombocytocrit	0.13 (0.06)	0.15 (0.08)	.51	0.258	0.18 (0.11)		0.17 (0.09)	.89	0.100
Activated partial thromboplastin time (s)	53.44 (10.79)	63.41 (37.08)	.33	0.155	42.87 (11.57)		48.39 (11.67)	.26	0.257
Prothrombin time (s)	23.71 (7.09)	25.63 (13.09)	.62	0.023	18.83 (4.74)		21.14 (8.5)	.42	0.166
Thrombin time (s)	33.51 (33.21)	22.25 (7.1)	.21	0.501	21.56 (3.53)		25.20 (10)	.25	0.241
Fibrinogen (g/L)	1.32 (0.5)	1.40 (0.67)	.70	0.030	2.16 (1.45)		1.73 (0.73)	.37	0.232
International normalized ratio	3.21 (4.44)	2.33 (1.32)	.47	0.235	1.64 (0.43)		1.85 (0.79)	.43	0.165
Uric acid (μmol/L)	228.38 (140.69)	239.09 (127.74)	.84	0.175	354.37 (143.16)		271.76 (143.86)	.18	0.695
Creatinine (μmol/L)	109.40 (69.88)	109.78 (75.06)	.99	0.028	131.90 (109.8)		152.6 (138.22)	.70	0.284
Urea (mmol/L)	8.10 (8.8)	9.75 (8.51)	.62	0.713	8.83 (10.98)		11.27 (9.87)	.59	0.672
Glucose (mmol/L)	8.47 (3.63)	6.41 (3.17)	.13	0.605	6.87 (1.73)		5.84 (1.85)	.19	0.364
Alanine aminotransferase (U/L)	689.29 (966.99)	50.58 (26.83)	.02	3.480	601.21 (1109.7)		247.04 (589.4)	.36	1.194
Aspartate aminotransferase (U/L)	593.71 (536.28)	76.61 (29.5)	.01	3.353	419.21 (570.33)		310.00 (525.12)	.64	1.146
Total bilirubin (μmol/L)	375.23 (193.41)	220.75 (152.33)	.02	1.280	205.73 (166.14)		268.68 (221.29)	.45	0.537
Conjugated bilirubin (μmol/L)	245.40 (146.5)	105.27 (68.85)	.01	2.062	136.83 (134.83)		174.74 (163.26)	.56	0.546
Unconjugated bilirubin (μmol/L)	120.25 (94.92)	104.04 (97.16)	.65	0.755	67.41 (55.92)		89.22 (99.74)	.53	0.086
Cholinesterase (U/L)	3736.25 (1493.02)	3027.83 (771.09)	.11	0.343	4107.52 (2203.11)		3282.87 (1256.68)	.30	0.257
γ-glutamyl (U/L)	192.02 (224.2)	105.41 (122.09)	.38	1.121	341.4 (324.48)		208.43 (193.01)	.39	0.518

^a^QD: quantitative difference.

### Assessment of the Therapeutic Efficacy of Liver Failure

**Figure 4:**
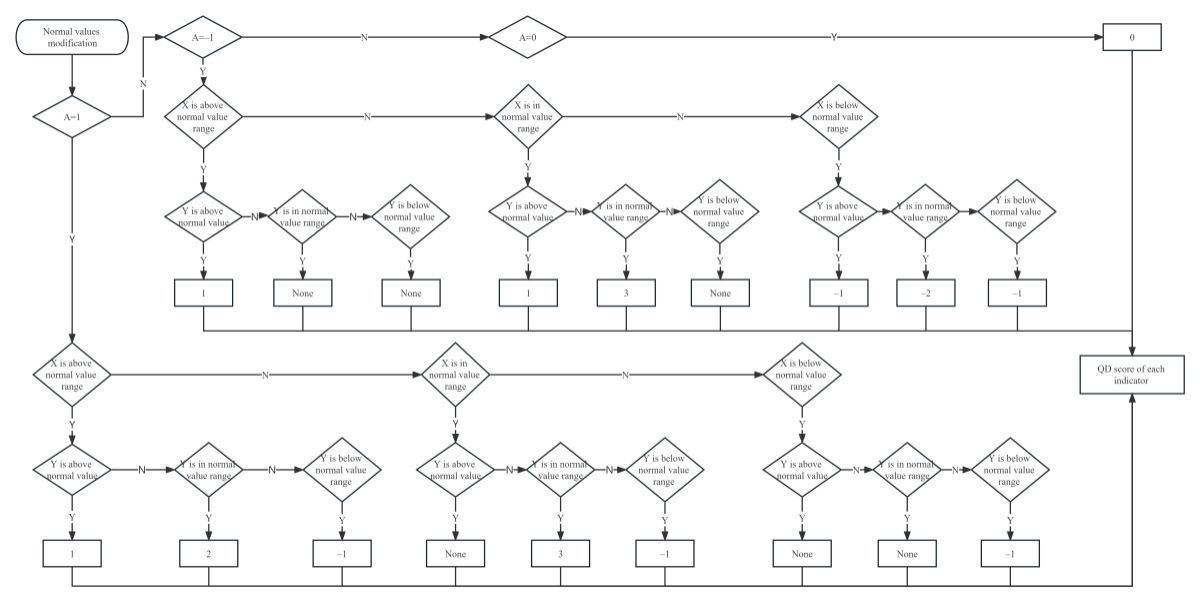
The detailed procedure of Part III.

Next, our objective is to use the QD algorithm to assess the effect of different treatments on ACLF and try to provide a novel approach to prognostic evaluation. The algorithm’s steps are outlined in Figures 3 and 4. Figure 3A provides an overview of the QD scoring system, while Figure 3B, Figure 3C, Figure 4, and Figure 3D elaborate on the detailed procedures for part 1, part 2, part 3 and part 4 in the scoring system, respectively. The procedure of the QD scoring system strictly adheres to the sequence outlined in Figure 3A.

Figure 3B illustrates the comprehensive procedure for comparing each parameter’s X and Y values. If the data after treatment are larger than the data before treatment, the algorithm assigns an output value of –1. If the data after treatment are smaller than the data before treatment, the output value is set to 1. The output value for this step is denoted as “A.”

Figure 3C provides a detailed procedure for calculating each parameter’s value. The indexes mentioned above for each patient in both the DPMAS and conventional treatment groups were collected before and after the treatments. The maximum value of a specific index is divided by the minimum value of the same index before and after the therapy. Then, we calculated the golden logarithm of the value and set it as Lτ. When Lτ≤0.80, the output value is 0; when 0.80<Lτ≤1.22, the output value is 1, when 1.22<Lτ≤1.69, the output value is 2; and when Lτ>1.69, the output value is 3. The output value for this step is denoted as “B.”

Figure 4 elaborates on the detailed procedure for modifying the normal values. This step involves the correction of the range of normal values. Although the first 2 steps allow for assessing the direction and magnitude of changes before and after treatment, they do not consider whether these changes represent an improvement or deterioration in the patient’s condition. Hence, this step is used to evaluate patient index changes. The output value for this step is denoted as “C.”

Figure 3D indicates the detailed procedure for modifying the 4 liver function indicators. The scoring system calculates the QD score for each indicator by multiplying the values obtained from the previous steps (A, B, and C). After obtaining the QD scores for each indicator, the scoring system proceeds with the modification of 4 liver function indicators (ie, alanine aminotransferase, aspartate aminotransferase, total bilirubin, and conjugated bilirubin). After analyzing the data, we found the 4 indicators of healed patients had significantly decreased after treatments, proven by the *P* value and the QD algorithm. However, the patients who dropped out or died only had 2 significantly reduced markers (alanine aminotransferase and aspartate aminotransferase). First, the changes in the 4 indicators (ie, alanine aminotransferase, aspartate aminotransferase, total bilirubin, and conjugated bilirubin) needed to be examined by evaluating their respective A values. If the sum of them equaled 4, all 4 indicators decreased after the intervention. The output of this assessment is denoted as the D value. Then, we needed to examine whether the B value of the 4 indicators was ≥9, ensuring that at least 3 indicators significantly decreased after the treatment. The result of this examination is denoted as the E value. Finally, we calculated the sum of the scores for all indicators (except alanine aminotransferase, aspartate aminotransferase, total bilirubin, and conjugated bilirubin) as well as the score obtained from the modification of the 4 liver function indicators. A higher score for each patient indicates better therapeutic efficacy.

**Figure 5:**
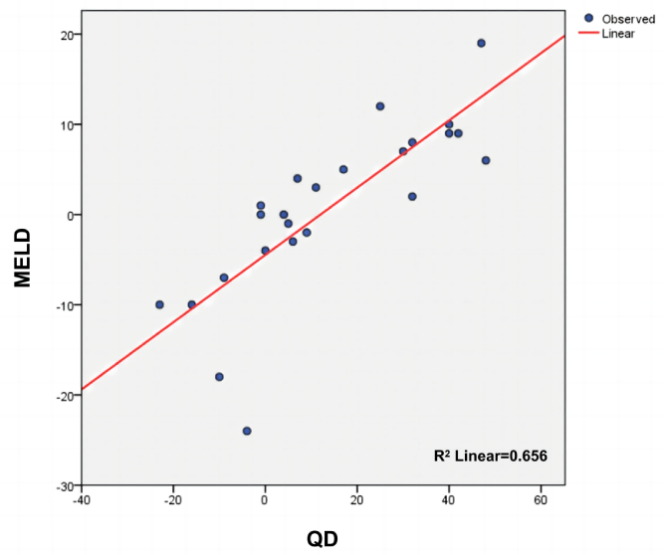
Linear regression models. The correlations between the Model for End-Stage Liver Disease (MELD) score and the quantitative difference (QD) algorithm score are shown.

According to the procedures mentioned above, we calculated the QD score for each patient. We compared scores that were calculated by 2 different scoring systems, and there was a significant association between MELD score and the QD score (*P*<.001; Figure 5).

Next, we compared each patient’s clinical status before and after treatment and tried to find the correlation between clinical status and QD scores. We found that in patients whose fatigue had improved, their QD scores were significantly higher than those of patients whose clinical status had deteriorated or remained unchanged (*P=*.006; Table 3).

**Table 3:** Clinical status and quantitative difference (QD) algorithm score data.

Clinical state	Improved, n	QD score, mean (SD)	Deteriorated or unchanged, n	QD score, mean (SD)	*P* value
Fatigue	10	28.50 (21.25)	8	1.25 (13.33)	.01
Abdominal distension	8	18.00 (17.44)	9	6.33 (18.71)	.21
Anorexia	8	26.63 (23.46)	6	5.83 (10.53)	.07
Jaundice	11	19.64 (22.46)	7	3.43 (12.05)	.10
Oedema of lower limb	4	20.75 (20.98)	2	–0.50 (0.71)	.25

**Figure 6:**
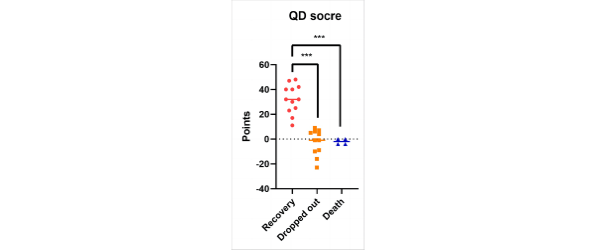
The quantitative difference (QD) algorithm score of patients' different statuses after therapy. Data are presented as mean (SD).

Next, we divided patients into 3 groups according to patient status to verify whether the QD scoring system could reflect postoperative patient status. We found that the QD scores of improved patients were significantly greater than those who had dropped out or died (*P<*.001; Figure 6). The calculation table of the QD algorithm scoring system is presented in Multimedia Appendix 1.

## Discussion

### Principal Findings

The prognosis evaluation of liver failure should run through the entire diagnosis and treatment process. However, it is difficult to objectively evaluate the therapeutic effect of ACLF because of the complex progress of liver failure and multiple impact factors. Although most of the prognostic models in hepatology were developed as static models, the full predictive potential of the dynamic trajectory of these models has received little attention so far [20]. In this study, we introduced a novel model for liver failure prognosis evaluation based on the characteristics of the QD algorithm by comparing data from patients who received DPMAS or conventional treatment to evaluate the therapeutic dynamic. After calculating the QD score of each patient, a significant correlation was found between the MELD score and the QD score (*P*<.001), substantiating that the QD scoring system can effectively gauge the therapeutic effects in patients with ACLF, akin to the MELD scoring system. Next, we compared the clinical status of patients with their QD scores. Improvement of fatigue showed a significant correlation in our study (*P=*.006). The QD score of the recovery group was significantly higher than that of the patients who dropped out of therapy and the death group (*P*<.001), indicating that the QD scoring system can effectively reflect the patient’s status after treatment.

Liver failure is associated with increased metabolites and toxins, such as bilirubin, ammonia, glutamine, aromatic amino acids, and proinflammatory cytokines [33-35]. These toxins are known to play an essential role in the pathogenesis of liver failure [36-40]. Studies on artificial liver have identified significant reductions in serum bilirubin, urea, and creatinine levels in patients with ACLF [39-41]; this improvement in survival rates is attributed to the clearance of ammonia and nitrogen-carrying molecules, such as glutamine and alanine. Total bilirubin and conjugated bilirubin are reduced, whereas no changes in unconjugated bilirubin levels are observed [42]. We found significant differences in alanine aminotransferase, aspartate aminotransferase, and conjugated bilirubin in both *P* values and QD values in the DPMAS group. These findings of the abovementioned studies closely align with the results of our study, which confirmed that the chosen threshold in the QD algorithm was reasonable.

As a fixed algorithm, the MELD scoring system was initially developed to objectively determine the priority of liver transplantation and predict short-term mortality in patients with liver disease. It was built using only subjective parameters. Later, a vast body of research demonstrated its prognostic ability, and it continues to maintain the characteristics of the MELD scoring system by using subjective parameters and short-term mortality as prognostic indicators. In this context, the QD algorithm offers a novel way to dynamically evaluate the therapeutic effects in each patient instead of using a fixed algorithm like MELD. Researchers and clinicians can input data from patients into the QD algorithm to obtain the QD score, which can be used to verify therapy efficacy and achieve the objectives of the analysis. Of note, individual variability may contribute to the high SDs observed in the QD scores.

### Limitations

Our study has limitations. The sample size was relatively small, and the follow-up period was short. It should be emphasized that trials of DPMAS are difficult to perform and control appropriately for several reasons, including a lack of well-characterized patients and heterogeneity of causes.

### Conclusions

In conclusion, the QD scoring system can measure the therapeutic effects in patients with ACLF, similar to the MELD scoring system, but surpasses it by incorporating a broader range of indicators and considering patient variability. The QD algorithm can pave the path of tailoring treatment by comparing the difference between pre- and posttreatment for the same patients, which may lead to more precise and effective interventions for patients with ACLF. Future work is needed to assess whether the proposed algorithm applies to other liver diseases, calling for a larger data set and additional samples for clinical validation.

## Appendix

Multimedia Appendix 1 The calculation table of the quantitative difference (QD) algorithm scoring system.

## Abbreviations

ACLF: acute-on-chronic liver failure
DPMAS: double plasma molecular absorption system
MELD: Model for End-Stage Liver Disease
QD: quantitative difference

## Data Availability

The data sets generated and analyzed during this study are available from the corresponding author on reasonable request.
